# Laparoscopic repair of a traumatic diaphragmatic rupture

**DOI:** 10.1016/j.ijscr.2024.109644

**Published:** 2024-04-20

**Authors:** Cedric M.W. Pesch, Shiromani Janki, Dashti Faraj, Willem E. Hueting

**Affiliations:** a Groene Hart Ziekenhuis, Gouda, Netherlands; b LUMC, Leiden, Netherlands; c Alrijne Ziekenhuis, Leiderdorp, Netherlands

**Keywords:** Diaphragm, Blast injury, Chronic disease, Laparoscopy, Case reports

## Abstract

**Introduction and importance:**

Traumatic diaphragmatic ruptures following blast injury or penetrating trauma rarely present themselves with chronic symptoms warranting elective surgery.

**Case presentation:**

We present the case of a 49-year-old man who survived a grenade explosion and experienced chronic chest pain. Considering the previous trauma, computed tomography imaging was performed and showed a left-sided traumatic diaphragmatic rupture ventral to the spleen, resulting in herniation of the transverse colon and omentum in the thoracic cavity. Metal shrapnel was located between the stomach and spleen, the suspected cause of the diaphragmatic hernia. The patient was eligible for minimal invasive laparoscopic surgery.

**Clinical discussion:**

During surgery, a left diaphragmatic rupture and metal shrapnel on the right side of the rupture were found. The hernia was reduced and the metal shrapnel was removed, aiding in fully repositioning of the omentum and transversed colon. After which the left lower lung lobe was able to fully inflate. The rupture was closed using single V-lock sutures and strips of the Phasix mesh to reinforce the diaphragm repair with single ethibond sutures. No surgical or post-operative complications were observed and the patient did not experience any of his previous complaints.

**Conclusion:**

In this case, laparoscopic repair of diaphragmatic rupture after penetrating trauma can be considered as an effective surgical approach.

## Highlights

• Laparoscopic repair of penetrating traumatic diaphragm ruptures is effective after delayed presentation.
• Penetrating diaphragm trauma is rare and tends to be small and can be missed in acute settings
• Minor trauma to the diaphragm creates an opportunity to plan minimal invasive surgery

## 1 Introduction

Diaphragmatic rupture following blunt or penetrating trauma is uncommon in blast injuries to the thorax or abdomen and is present only in 0.5 % of all trauma cases. When present, some cases need immediate treatment when patients remain hemodynamically unstable due to bleeding, experience respiratory problems or have incarcerated organs. In the acute phase, radiological reports correctly diagnose diaphragmatic ruptures in 40 % of left sided diaphragm injuries [1,2]. If immediate treatment is mandatory, the treatment of choice often was open surgery [14]. However, recent literature findings state that laparoscopic repair is just as good or even better because of increased per operative visibility and decreased post-operative pain when compared to open surgery [3]. Most of these findings include acute trauma care and little is found on patients who experience symptoms at a later stage [4]. Due to blast injury the location of the diaphragmatic rupture may vary, and therefore presentation of symptoms may vary. When comparing blunt with penetrating trauma as cause of a diaphragmatic rupture, the latter often offers an opportunity to wait for the patient to heal and perform elective minimally invasive surgery [5]. Traumatic diaphragmatic rupture can also remain unnoticed and develop symptoms at a later stage, which symptoms are more similar to congenital diaphragmatic hernia, such as coughing, reflux, thoraco-abdominal discomfort, dyspnea or dysphagia. Our case report describes this more rare situation.

## 2 Case report

In this case report, constructed in line with SCARE criteria [13], we present a 49-years-old Ukrainian patient with a history of obstructive sleep apnea syndrome (OSAS) with use of continuous positive airway pressure (CPAP), hypertension and conchotomy, who had survived a blast injury following a car explosion in Iraq in 2006. Except one laceration on the left lateral side of the abdomen, the patient experienced no other injury. After fleeing the current war in Ukraine, the man presented himself as a patient in our outpatient clinic in 2022 due to chronic pain on the left ventral side of the thorax which he could clearly locate and which increased with physical activity.

**Fig. 1:**
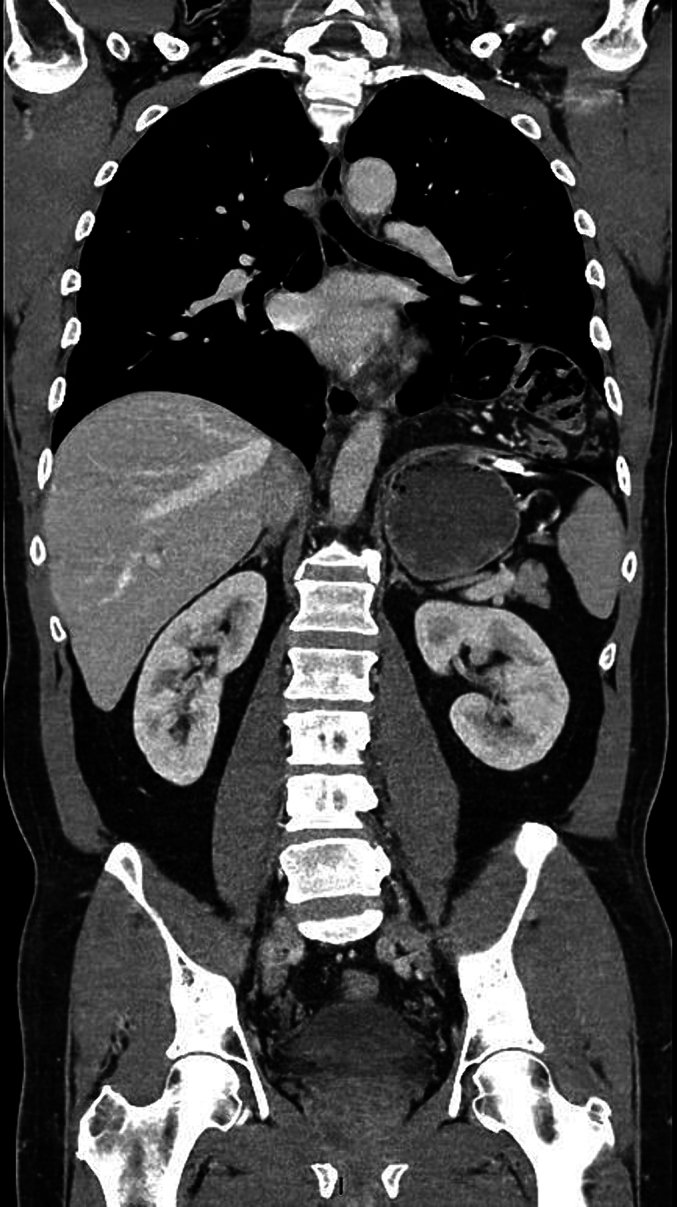
CT-thor/abd. with IV contrast, coronal cross-section

**Fig. 2:**
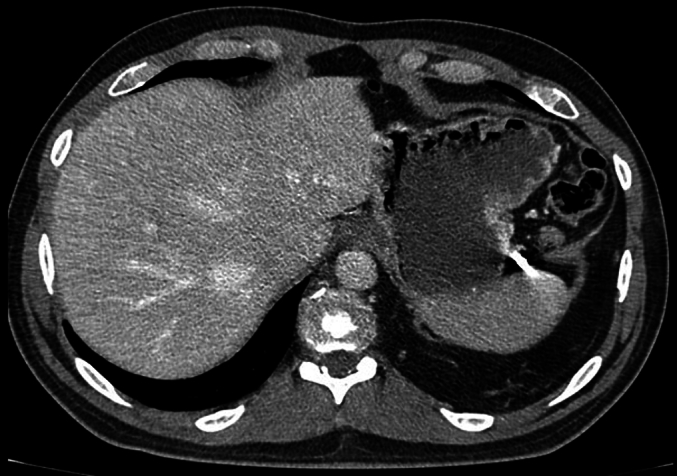
CT-thor/abd. with IV contrast, transverse cross-section.

Physical examination showed a healed laceration on the left flank in the mid-axillary line with normal breath sounds. Computerized tomography with contrast fluid demonstrated: a diaphragmatic rupture of 3 cm ventral to the spleen, part of the transverse colon was herniated into the thoracic cavity without signs of incarceration, metal shrapnel was detected in the abdominal cavity just below the diaphragm between the stomach and spleen and right near the diaphragmatic rupture, and atelectasis of the lower lobe of the left lung was suspected as well (Fig. 1, Fig. 2). The patient was diagnosed with a traumatic penetrating diaphragmatic rupture. Due to the absence of incarceration of the transverse colon and chronic nature of the symptoms the patient was eligible for elective minimal invasive surgery.

**Fig. 3:**
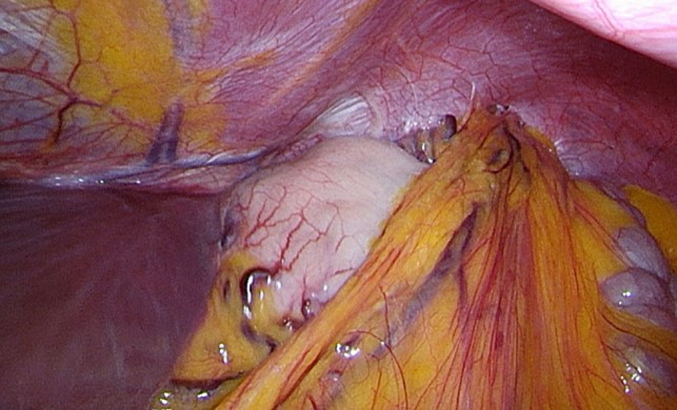
Intra-abdominal view of diaphragmatic rupture and intra-thoracic positioned colon and omentum.

**Fig. 4:**
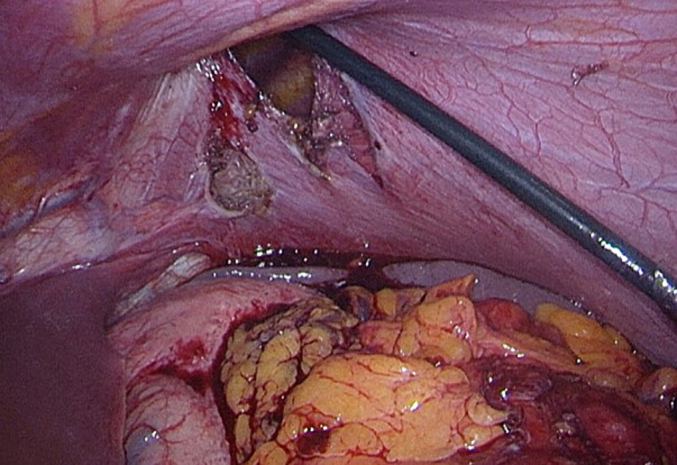
Intra-abdominal view of repositioned transverse colon and omentum.

**Fig. 5:**
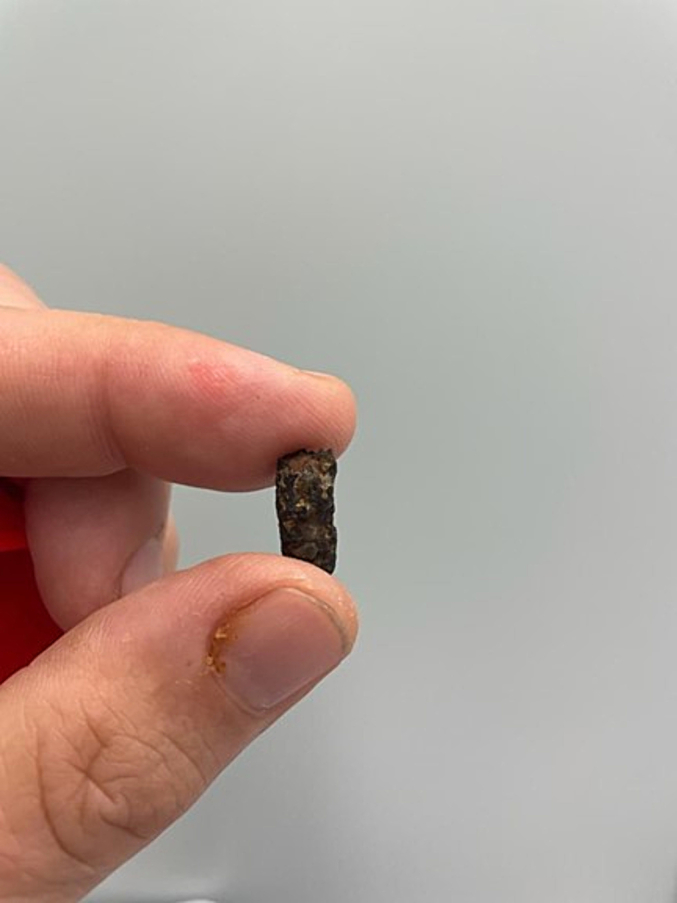
Metal shrapnel of grenade removed from right side of diaphragmatic rupture.

Laparoscopic surgery was performed eleven months after the diagnosis (by DF and WH). Antibiotic prophylaxis was administered before surgery, as is standard of care. Upon surgical inspection, a left-sided rupture of the diaphragm was seen with the shrapnel on the right edge of the rupture. The hernia sac was resected in small steps and the remnant metal shrapnel was removed, resulting in complete reposition of the transverse colon and accompanying omentum (Fig. 3, Fig. 4, Fig. 5).

**Fig. 6:**
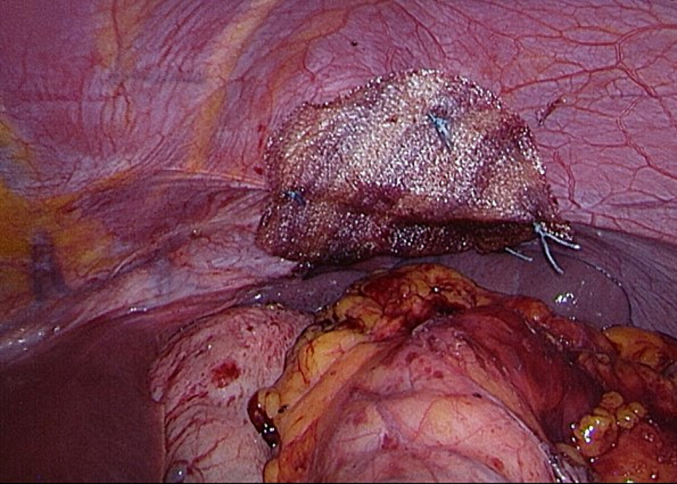
Intra-abdominal view of repaired diaphragmatic rupture with Phasix™ ST Mesh.

Inspection of the thoracic cavity demonstrated atelectasis of the lower left lobe without injury. After administration of positive end-expiratory pressure (PEEP) by the anesthesiologist the left lower lung lobe was able to be fully inflated. The diaphragmatic rupture was then closed from the dorsal side with interrupted non-absorbable V-lock sutures, after which the mesh (Phasix™ ST Mesh, BD, Vaud, Switzerland) was placed to reinforce the diaphragm. The rough part of the Phasix mesh was placed against the diaphragm and sutured with single sutures (Ethibond; Ethicon Inc., Somerville, NJ, USA) to cover the closed rupture. The mesh was placed in the following steps: first, the suture was pulled through the strip, then through the left side of the rupture and back through the right side of the rupture and back through the mesh again. The right tension of the reinforcement was obtained by tactile feedback [6] (Fig. 6).

Trocarts were removed under direct sight and a sign-out procedure was performed, concluding the laparoscopic correction of a traumatic diaphragmatic rupture and removal of grenade shrapnel of the left diaphragm, reposition of the colon transversum and omentum and closing the diaphragm with the use of a Phasix mat. No significant blood loss was observed and operating time did not exceed the time compared to an elective diaphragm hernia repair.

After surgery, dipidolor, eskatamine, clonidine and a lidoperfusor were administered because of pain in the operation area. The history of OSAS, the administration of pain medication and the fact that the patient did not bring his CPAP mask to the hospital, warranted the need for observation in the ICU per hospital protocol. No abnormalities were observed during the ICU stay after which the patient was transferred to the ward the next day. Two days after surgery the patient was discharged with oral analgesics and outpatient appointments were scheduled in two and six weeks.

During follow up the patient showed no signs of post-operative complications. The abdominal wounds had healed well and the patient was able to perform physical activity without pain. No problems were experienced with digestion. After 6 weeks the patient was discharged from the outpatient clinic.

Ten months after surgery, the patient experienced pain in the left flank again and was seen by one of the surgeons (WH). No abnormalities were found during physical examination and a CT-scan was performed and compared to the first CT-scan. No abnormalities were seen besides a slightly elevated left diaphragm; no signs of residual hernia or any abdominal or thoracic pathology. The patient was reassured that no surgical complication had occurred.

## 3 Discussion

Penetrating diaphragm trauma is rare and usually causes smaller injuries (< 2 cm) when compared to blunt trauma [7]. As a result, the diagnosis can be missed in the acute setting either due to other injury or due to the small injury of these diaphragmatic ruptures which can sometimes be asymptomatic. Only after changes in haemodynamics, due to incarceration or obstruction, or the presence of non-specific symptoms like chest pain or dyspnea the diagnosis will become more apparent [4].

The literature mostly consists of other case-reports describing diaphragmatic ruptures caused by penetrating and blunt trauma in the acute setting. However, the clinical presentation can vary in onset as has been described by other case reports.

There is no formal guideline on the ideal surgical approach to repair delayed diaphragmatic ruptures. In a retrospective analysis, 40 patients with delayed diaphragmatic ruptures were analyzed. The onset of symptoms differed between 4 months to 21 years. Of 36 patients the mechanism of injury was known; 21 patients suffered from blunt trauma and 15 from penetrating trauma. The preferred operation technique was a thoracotomy in 38 of 40 patients, which is still the golden standard in many hospitals. The rationale for this is threefold; the absence of acute or injury to the abdominal cavity makes laparotomy inferior to thoracotomy. Furthermore, adhesions of intestinal- and lung tissue and viscera are much more likely to be present due to the delayed diagnosis which could make laparoscopic repair more difficult. Lastly, not much literature was previously available on the laparoscopic approach on delayed diaphragmatic rupture repair [9].

The shifted anatomy and presence of adhesions create a risk of tension pneumothorax or cardio-respiratory problems when laparoscopically retrieving and resecting the hernia sac [10]. However, minimal invasive surgery is an elegant approach and has many advantages such as a smaller wound surface avoiding blood loss, pain and discomfort and decreases the chance of infection and wound dehiscence [11]. Over the last decennium, more literature has become available describing laparoscopic repair of chronic traumatic diaphragmatic hernias. In a collection of 23 cases, laparoscopic repair of traumatic chronic diaphragmatic rupture was found to be effective, leading to fast recovery, high security and effectiveness; mean blood loss was 63.48 ± 71.69 ml and postoperative hospital stay ranged from 5 to 15 days [15]. Rashid et al. described one case after blunt trauma that had occurred 9 years ago during a fall without any visible injury. Later on, the patient developed abdominal pain and nausea which led to multiple admissions without being able to find the cause of the symptoms. No change in hemodynamics occurred and only after performing a CT-scan the diaphragmatic rupture with an intrathoracic colon was seen. The rupture was repaired laparoscopically with Gortex sutures and a porcine mesh, resulting in full recovery [8]. The laparoscopic approach has also proven to be effective in case of recurrent diaphragmatic hernia after previously treated traumatic diaphragmatic ruptures. Singh et al. reported a case of a 23-year-old male who suffered from a recurring left-sided diaphragmatic hernia due to a diaphragmatic rupture caused by gun-shot injury. The initial rupture was treated by a combined thoracotomy and laparotomy 2 years prior to presentation. The laparoscopic repair was performed successfully by using a polypropylene mesh and 2-0 polypropylene suture, resulting in full recovery [12]. Minimal invasive surgery has risen to one of the most used and appreciated methods of surgery, but has not reached its full potential in the world of trauma surgery.

## 4 Conclusion

This case report demonstrates and contributes to the fact that diaphragmatic rupture caused by penetrating trauma, without signs of incarceration, is eligible for elective minimal invasive surgery at a later stage. Usually these types of traumatic injury require immediate care, limiting the time between diagnosis and treatment. However, in minor trauma cases, as described in this case report, the delayed diagnosis creates time to perform adequate additional imaging and planning which provides the opportunity of elective minimal invasive surgery.

## Informed consent

Written informed consent was obtained from the patient for publication of this case report and accompanying images. A copy of the written consent is available for review by the Editor-in-Chief of this journal on request.

## Ethical approval

Ethics clearance was not necessary; every treatment-related action was performed according to standard of care, no deviation of standard protocol took place, no unlicensed product was used and decision to publish data was made after actual patient treatment.

## Funding

No funding sources were used in the creation and publishing of this case report.

## Author contribution

CP: data collection, data analysis, writing the paper

SJ: data analysis, writing the paper, reviewing the paper

WH: reviewing the paper

DF: reviewing the paper

## Guarantor

Willem Hueting, MD PhD

Dashti Faraj, MD

## Declaration of competing interest

C. Pesch: no conflict of interest.

S. Janki: no conflict of interest.

W. Hueting: no conflict of interest.

D. Faraj: no conflict of interest.
